# Japanese Encephalitis Virus as Cause of Acute Encephalitis, Bhutan

**DOI:** 10.3201/eid2609.200620

**Published:** 2020-09

**Authors:** Sonam Wangchuk, Tshewang Dorji Tamang, Jit Bahadur Darnal, Sonam Pelden, Karma Lhazeen, Mimi Lhamu Mynak, G. William Letson, Shalini Khare, Brandon Troy Leader, Anthony A. Marfin, Susan L. Hills

**Affiliations:** Royal Centre for Disease Control Thimphu, Bhutan (S. Wangchuk, J.B. Darnal, S. Pelden);; Department of Public Health, Thimphu (T.D. Tamang, K. Lhazeen);; Jigme Dorji Wangchuk National Referral Hospital, Thimphu (M.L. Mynak);; PATH, Seattle, Washington, USA (G.W. Letson, B.T. Leader, A.A. Marfin);; PATH India, Delhi, India (S. Khare);; Centers for Disease Control and Prevention, Fort Collins, Colorado, USA (S.L. Hills)

**Keywords:** Japanese encephalitis, vector-borne disease, Bhutan, acute encephalitis syndrome, meningitis/encephalitis, vector-borne infections, mosquitoes, viruses

## Abstract

In 2011, Bhutan’s Royal Centre for Disease Control began Japanese encephalitis (JE) surveillance at 5 sentinel hospitals throughout Bhutan. During 2011–2018, a total of 20 JE cases were detected, indicating JE virus causes encephalitis in Bhutan. Maintaining JE surveillance will help improve understanding of JE epidemiology in this country.

Japanese encephalitis virus (JEV), a mosquitoborne flavivirus, is a common cause of encephalitis in Asia (*1*). Japanese encephalitis (JE) causes considerable illness and death, particularly in children <15 years of age (*2*). No specific treatment exists, but JE is preventable by vaccination.

JEV is maintained in an enzootic cycle between mosquitoes and amplifying vertebrate hosts, primarily pigs and wading birds (*2*). *Culex* mosquitoes are the principal vectors, especially *Cx. tritaeniorhynchus*, and commonly breed in rice fields and other stagnant water collections (*2*). JEV transmission occurs predominantly in rural agricultural areas (*2*).

In Bhutan, JEV vectors are prevalent in many southern districts and in some interior districts. Five *Culex* mosquito species have been identified: *Cx. tritaeniorhynchus*, *Cx. vishnui*, *Cx. pseudovishnui*, *Cx. gelidus*, and *Cx. quinquefasciatus.* In particular, *Cx. tritaeniorhynchus* mosquitoes have been documented in the southern districts of Chukha, Samtse, Sarpang, and Samdrup Jonghkar. In much of the country, rice fields and other mosquito breeding sites are common (G.M. Yeshey et al., unpub. data, https://www.researchgate.net/publication/277224776_Effect_of_mineral_fertilizers_on_rice_productivity_in_Punakha-Wangdue_Valley), and pigs and wading birds can be found. At least 18,800 pigs were reported in Bhutan in 2017 and reared in centralized government breeding farms, with up to several hundred pigs, or in backyard farms, typically with <5 pigs (*3*,*4*). About two thirds of the country’s »750,000 persons live in rural areas (*5*). In consideration of the favorable conditions for JEV transmission and proximity to other JE-endemic countries, in 2011, the Royal Centre for Disease Control, Ministry of Health, implemented surveillance to investigate JE presence among humans in Bhutan.

## The Surveillance

Bhutan’s landscape ranges from lowland plains in the south to the Himalayan mountains in the north (*6*). The climate varies with elevation: very cold year-round in the north, temperate in the midlands, and subtropical in the south. Monsoon season spans mid-July through September. Bhutan has 20 administrative districts each with >1 general hospital. The regional referral hospital in Sarpang district in the south serves the central region and the referral hospital in Mongar district in the east serves the eastern region. The national referral hospital in the capital Thimphu also serves as the regional referral hospital for the western region. 

The Royal Centre for Disease Control has conducted sentinel site–based JE surveillance at 5 sites since 2011: the national and 2 regional referral hospitals, Phuntsholing hospital in Chukha district in the southwest, and Samdrup Jongkhar hospital in Samdrup Jongkhar district in the southeast (Figure). The Royal Centre for Disease Control staff based surveillance case definitions on those from the World Health Organization (WHO) JE surveillance standards (*7*). A clinical acute encephalitis syndrome (AES) case is illness in a person with acute onset of fever and >1 of the following: a change in mental status or new onset of seizures (excluding simple febrile seizures). A JE case is illness in a person with AES and laboratory evidence of JEV infection through detection of JEV IgM in serum or cerebrospinal fluid.

**Figure:**
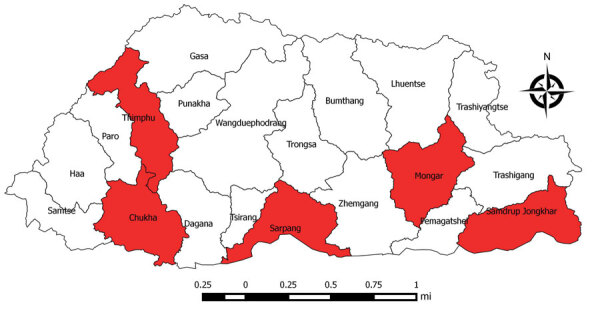
District locations of Japanese encephalitis sentinel surveillances sites (red shading), Bhutan.

When an AES case is identified at a sentinel site, clinicians collect serum or cerebrospinal fluid samples from the affected person and send them to the Royal Centre for Disease Control laboratory for testing using JE IgM-capture ELISA; WHO provides the serologic assays (*8*). Since 2017, testing for dengue virus IgM in serum also has been conducted (*8*). Because the Royal Centre for Disease Control staff gathered data as part of routine public health surveillance activities, institutional review board review was not required.

We calculated incidence using Bhutan census annual estimates (*9*). Because the catchment population sizes of each sentinel hospital were unavailable, we could not calculate precise incidence estimates, and we based estimates on national population data.

During 2011–2018, among 680 AES patients for whom samples were tested, 20 (3%) had JEV infection based on IgM detection in serum (n = 15) or cerebrospinal fluid (n = 5). An annual median of 2.5 (range 0–5) JE cases were detected (Table). In 2017, after serum testing began for dengue, no dengue virus IgM was found in the 1 JEV IgM-positive patient tested. The regional referral hospital in Sarpang district reported 2 cases; the regional referral hospital in Mongar district, 4 cases; and the national referral hospital in Thimphu, 14 cases.

**Table:** Japanese encephalitis cases and incidence based on sentinel surveillance at 5 hospitals, Bhutan*

Cases and incidence	2011	2012	2013	2014	2015	2016	2017	2018
No. cases	3	0	2	2	5	4	3	1
Incidence/100,000 population*	0.4	0	0.3	0.3	0.7	0.5	0.4	0.1
No. cases in children <15 y	3	0	2	1	4	3	1	0
Incidence/100,000 children <15 y*	1.4	0	0.9	0.4	1.7	1.3	0.5	0

*Incidence calculations were based on the total population of Bhutan because sentinel hospital catchment population sizes were unavailable.

The median age of JE patients was 8.5 years (range 1.4–63.0 years). Fourteen (70%) cases were in children <15 years of age; in this age group, the median age was 5.8 years. The overall male:female ratio was 1:0.7; however, for children <15 years of age, the ratio was 1:1.3. The average annual incidence during the 8-year period was 0.3 (range 0–0.7) JE cases/100,000 population, and for children <15 years, 0.8 (range 0–1.7) cases/100,000 (Table).

## Conclusions

During 2011–2018, sentinel site–based surveillance detected 20 JE cases, indicating JEV as a cause of encephalitis in Bhutan. Similar to JE epidemiology in many other Asian countries, most (70%) cases occurred among children <15 years of age (*2*).

The average annual incidence estimates of 0.3 JE cases/100,000 total population and 0.8 cases/100,000 children <15 years of age most likely underestimate national disease incidence, because they are based on cases reported from 5 sentinel hospitals. These 5 hospitals are unlikely to capture JE cases from all of Bhutan’s 20 districts, despite being geographically widespread, including where JEV transmission is probably highest; incorporating the country’s 3 referral hospitals; and hospital staff reporting a high number of AES cases, suggesting good awareness of reporting requirements (*6*).

Our results are subject to limitations. Because cross-reactivity can occur between JEV and other flavivirus antibodies in serologic assays and no confirmatory testing was possible, >1 dengue or other flavivirus infection could have been misclassified as JEV infection. In addition, surveillance staff did not collect information about travel history; however, children, who represented 70% of all cases, are unlikely to have traveled to other JE-endemic countries.

To better elucidate JE epidemiology and refine incidence estimates, sentinel surveillance needs further strengthening, including possibly increasing the number of sites, improving epidemiologic data completeness, gathering patient outcome information, ensuring testing of both cerebrospinal fluid and serum samples whenever possible, and facilitating confirmatory testing at a reference laboratory. Collection of place of residence also would be useful, although most patients most likely reside in southern districts, where more JEV vectors are present.

Evidence of JEV transmission in Bhutan is not surprising, given the country’s geographic location. JEV transmission has long been recognized in the bordering Indian states of Assam, West Bengal, and Arunachal Pradesh, which share similar ecologic conditions to southern and eastern Bhutan (*10*–*12*). All 3 Indian states have already established JE vaccination programs (*12*,*13*).

WHO recommends integration of JE vaccination into national immunization programs where JE is a public health priority. If case numbers are low, vaccination should be considered in areas with suitable animal reservoirs, ecologic conditions for transmission, and proximity to other JE-endemic countries (2). A vaccination program’s costs and benefits should be considered (*14*); of the 3 WHO-prequalified JE vaccines, >1 is considered affordable for use in lower income countries (*1*).

Our findings will assist decision-making on JE vaccine introduction in Bhutan. Maintaining AES and JE surveillance and ensuring complete data collection and sample testing will enable improved understanding of JE epidemiology.
